# Cancer cell-derived exosomal miR-425-3p induces white adipocyte atrophy

**DOI:** 10.1080/21623945.2022.2108558

**Published:** 2022-08-08

**Authors:** Anwen Liu, Wenxia Pan, Shutong Zhuang, Yuanzhi Tang, Haitao Zhang

**Affiliations:** Department of Gastrointestinal Surgery, Shenzhen Second People’s Hospital, Shenzhen, Guangdong, China

**Keywords:** Exosome, miR-425-3p, PDE4B, atrophy, adipocytes

## Abstract

White adipose tissue wasting plays a critical role in the development and progression of cancer cachexia. However, the mechanism behind the loss of adipose tissue remains ill-defined. In this study, we found that cancer cell-derived exosomes highly expressed miR-425-3p. Administration of cancer cell-derived exosomes significantly inhibited proliferation and differentiation of human preadipocytes-viscereal (HPA-v) cells. In mature adipocytes, cancer cell-derived exosomes activated cAMP/PKA signalling and lipophagy, leading to adipocyte lipolysis and browning of white adipocytes. These exosomes-induced alterations were almost abolished by endocytosis inhibitor cytochalasin D (CytoD) and antagomiR-425-3p, or reproduced by miR-425-3p mimics. In addition, bioinformatics analysis and luciferase reporter assay revealed that miR-425-3p directly targeted proliferation-related genes such as *GATA2, IGFBP4, MMP15*, differentiation-related gene *CEBPA*, and phosphodiesterase 4B gene (*PDE4B*). Depletion of PDE4B enhanced cAMP/PKA signalling and lipophagy, but had no effects on HPA-v proliferation and differentiation. Taken together, these results suggested that cancer cell-derived exosomal miR-425-3p inhibited preadipocyte proliferation and differentiation, increased adipocyte lipolysis, and promoted browning of white adipocytes, all of which might contribute to adipocyte atrophy and ultimately the loss of adipose tissue in cancer cachexia.

**Abbreviations:** ADPN: adiponectin; aP2: adipocyte protein 2 or fatty acid binding protein 4 (FABP4); BCA: bicinchoninic acid assay; BFA: bafilomycin A1; BMI: body mass index; C/EBP: CCAAT/enhancer binding protein; CEBPA: CCAAT/enhancer-binding protein-alpha; C-Exo: cancer cell-derived exosomes; CNTL: control; CREB: cAMP-response element binding protein; CytoD: cytochalasin D; ECL: chemiluminescence; GATA2: GATA Binding Protein 2; HFD: high fat diet; HSL: hormone-sensitive lipase; IGFBP4: insulin like growth factor binding protein 4; IRS-1: insulin receptor substrate-1; ISO: isoproterenol hydrochloride; KD: knockdown; KO: knock out; LC3: microtubule-associated protein 1A/1B-light chain 3; LMF: lipid mobilizing factor; LPL: lipoprotein lipase; MMP15: matrix metallopeptidase 15; Mir-Inh-C-Exo: cancer cell-derived exosomes with miR-425-3p inhibition; mTOR: mammalian target of rapamycin; Mut: mutant; N-Exo: normal cell-derived exosomes; NSCLC: non-small cell lung cancer; PBS, phosphate buffered saline; PGC-1: peroxisome proliferator-activated receptor-gamma coactivator-1; PDEs: phosphodiesterases; PKI: PKA inhibitor; PKA: cAMP-dependent protein kinase; PLIN1: Perilipin 1; PTHRP: parathyroid hormone-related protein; PVDF: polyvinylidene difluoride; shRNA: short hairpin RNA; UCP1: uncoupling protein 1; WT: wild type.

## Introduction

Cancer cachexia is a multifactorial, complex, destructive, and usually irreversible syndrome, characterized by a marked loss of body weight mainly induced by skeletal muscle wasting with or without progressive fat loss [1]. It affects about 50% to 80% of the patients with the advanced cancers such as non-small cell lung cancer (NSCLC), gastric cancer, colorectal cancer, and so on [1]. The patients with cancer cachexia suffer from a systemic functional impairment, increased treatment-related toxicity, poor quality of life, and reduced survival [1]. Although the animal study has evidenced that reversal of cancer cachexia can prolong the survival time of tumour-bearing mice [2], there are still lack of effective clinical therapies for the patients with cancer cachexia [3].

Growing evidence has indicated that, during cancer cachexia, the systemic and cellular metabolisms have undergone substantial changes, including glucose intolerance, increased protein catabolism in skeletal muscle, increased lipolysis in adipocytes, and browning or beige of white adipose tissues [3–5]. However, the specific mechanisms underlying these pathophysiological processes are required for more detailed explanations. It has been shown that cancer tissues or cells are responsible for the loss of skeletal muscle mass through affecting the protein and amino acid metabolisms, increasing cell apoptosis, and inhibiting regeneration of skeletal muscle cells [4]. Multiple signalling pathways including TGF-β, NF-κB, MAPK, PI3K/mTOR, and activin type-2 receptor (ActRIIB) have been suggested to be involved in the regulations of cancer-induced skeletal muscle wasting [4]. Meanwhile, tumour tissues or cells can also influence lipoprotein lipase (LPL) activity, lipid formation in adipose tissue (lipogenesis), fat breakdown, and browning or beige of white adipose tissues, through regulating lipid mobilizing factor (LMF), parathyroid hormone-related protein (PTHRP), IL-6 etc., respectively [4]. The synergistic impacts of these factors contribute to fat loss [5]. It is worth to note that white adipose tissue wasting occurs at an early stage of cancer cachexia, independently of the loss of skeletal muscle mass [6–8]. The functional restoration of white adipocytes in cancer cachexia models markedly ameliorates or prevents skeletal muscle wasting [7], suggesting the critical role of adipocytes in the development and progression of cancer cachexia. Therefore, it is of great significances to explore the molecular mechanisms behind adipocyte atrophy, which is a hallmark of cancer cachexia [9].

Various recent studies have confirmed the contributions of lipolysis and adipocyte browning in adipose atrophy. In cancer cachexia, adipocytes undergo increased lipolysis and reduced lipogenesis, independent of malnutrition. Thence, increased fat cell lipolysis has been considered as a primary cause of fat loss in the patients with cancer cachexia [10]. In addition to lipolysis, beige adipocyte induced by adipocyte browning stimulates high energy expenditure. Results from cell and animal models have demonstrated a strong correlation between adipocyte browning and adipose loss. However, the clinical evidence that beige adipocytes contribute to cancer cachexia is very limited, although brown adipocytes have recently been identified in adult humans. Nevertheless, blockade of lipolysis and/or adipocyte browning may clinically serve as potential targets for treatment of cancer cachexia [11].

Exosome is a kind of extracellular vesicles with a diameter of 30–100 nm. It can be secreted by multiple cell types and exists in different body fluids or culture medium. Functionally, exosome mediates cell-cell communication through its contents such as cellular proteins, lipids, RNAs (miRNAs, mRNAs, lncRNA), and DNAs [12]. Recent studies have suggested that cancer cell-derived exosomes are involved in the regulation of cell proliferation, angiogenesis, metastasis, chemotherapy resistance as well as immune escape [12,13]. Therefore, exosomes can be used to treat different cancers or act as novel therapeutic targets [12,13].

In the present study, we aimed to figure out the mechanisms by which cancer cells induce adipocyte atrophy. We found that cancer cell-derived exosomal miR-425-3p inhibited preadipocyte proliferation and differentiation through targeting proliferation- and differentiation-related genes. We also evidenced that exosomal miR-425-3p activated cAMP/PKA signalling and lipophagy, which was mediated by phosphodiesterase 4B (PDE4B), leading to an enhancement of adipocyte lipolysis and browning of white adipocytes. These findings bring about a novel mechanistic explanation for adipocyte atrophy in cancer cachexia and also hopefully provide a potential target in adipocytes for treating this wasting syndrome.

## Results

### Exosomal miR-425-3p highly expressed in cancer cells

**Figure 1.**
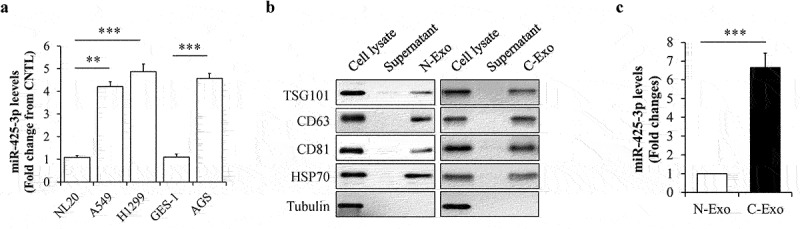
MiR-425-3p levels in cancer cell-derived exosomes. (a) Expression levels of miR-425-3p in cancer or normal cells (n = 4). (b) Identification of exosomes by its markers. (c) Expression levels of miR-425-3p in NL20 or A549 cell-derived exosomes (n = 6). Data are presented as mean ± SD; **p < 0.01, ***p < 0.001 *vs* indicated group. N-Exo: normal (NL20) cell-derived exosomes; C-Exo: cancer (A549) cell-derived exosome.

Previous study has documented that high levels of circulating exosomal miR-425-3p is positively associated with poor progression-free survival in the patients with NSCLC [14]. To figure out the sources of exosomal miR-425-3p, miR-425-3p levels in cachexia-inducing tumour A549, H1299, and AGS cells were compared with those in non-tumorigenic NL20 and GES-1 cells [15]. We found that cachexia-inducing tumour cells contained high levels of miR-425-3p (Figure 1(a)). Then, exosomes was isolated from culture medium and identified by its markers (Figure 1(b)). Through analysing miR-425-3p contents in exosomes by qRT-PCR, we confirmed that A549 cells secreted high levels of exosomal miR-425-3p, when compared with NL20 cells (Figure 1(c)). Thence, exosomes derived from A549 or NL20 cells were used for further study, representing cancer cell-derived exosomes (C-Exo) or normal cell-derived exosomes (N-Exo), respectively.

### Exosomal miR-425-3p inhibited preadipocyte proliferation and differentiation

**Figure 2.**
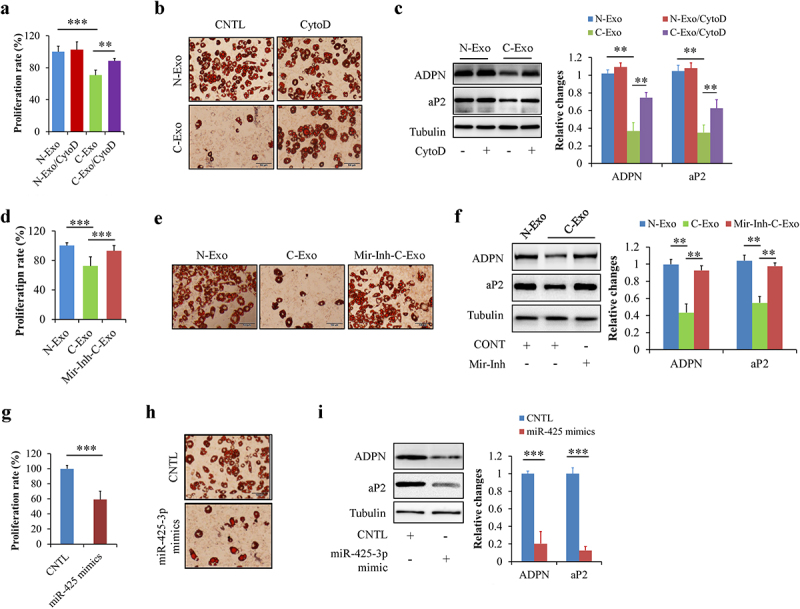
Exosomal miR-425-3p inhibited preadipocyte proliferation and differentiation. HPA-v cells were incubated with 50 μg/L of exosomes or 50 nM of miR-425-3p mimics for 24 h (for cell proliferation) or 7 days (for adipogenic differentiation), respectively. Adipocyte differentiation was induced using a standard protocol. CytoD (2 μg/mL) was administrated to inhibit endocytosis. Oil red O staining and western blot were performed at day 7 of adipogenic differentiation. (a) Effect of exosomes on cell proliferation. (b) Effect of exosomes on oil red O staining. (c) Effect of exosomes on the protein levels of aP2 and ADPN. (d) Effect of exosomes with miR-425-3p inhibition on cell proliferation. (e) Effect of exosomes with miR-425-3p inhibition on oil red O staining. (f) Effect of exosomes with miR-425-3p inhibition on the protein levels of aP2 and ADPN. (g) Effect of miR-425-3p mimics on cell proliferation. (h) Effect of miR-425-3p mimics on oil red O staining. (i) Effect of miR-425-3p mimics on the protein levels of aP2 and ADPN. Data are presented as mean ± SD, n = 4; **p < 0.01, ***p < 0.001 *vs* indicated group. N-Exo: normal (NL20) cell-derived exosomes; C-Exo: cancer (A549) cell-derived exosomes; Mir-Inh-C-Exo: cancer (A549) cell-derived exosomes with miR-425-3p inhibition; CytoD: cytochalasin D; aP2: adipocyte protein 2; ADPN: adiponectin; CNTL: control.

We firstly investigated the potential effects of N-Exo and CytoD on preadipocyte proliferation and differentiation. When HPA-v cells were incubated with 50 μg/L of N-Exo or 2 μg/mL of CytoD for 24 h, we found that neither N-Exo or CytoD affects cell proliferation and adipogenesis (Suppl. Figure S1a,b). To investigate the effects of exosomal miR-425-3p on preadipocyte proliferation and differentiation, HPA-v cells were incubated with 50 μg/L of C-Exo or Mir-Inh-C-Exo, an exosomes derived from A549 cells with miR-425-3p inhibition, in the presence or absence of 2 μg/mL of CytoD for 24 h. Equal concentration of N-Exo serviced as a normal control. CytoD acted as an endocytosis inhibitor to confirm the potential impacts of exosomes on cell proliferation and differentiation. We found that C-Exo treatment markedly reduced preadipocyte proliferation (Figure 2(a)) and differentiation (Figure 2(b,c)), when compared with N-Exo treatment. The similar alterations were observed in miR-425-3p mimics-treated cells (Figure 2(g-i)). Importantly, these C-Exo-induced changes were significantly mitigated by CytoD supplementation (Figure 2(a-c)) or by inhibition of miR-425-3p (Figure 2(d-f)). These results suggested that cancer cell-derived exosomal miR-425-3p suppresses preadipocyte proliferation and differentiation.

**Figure 3.**
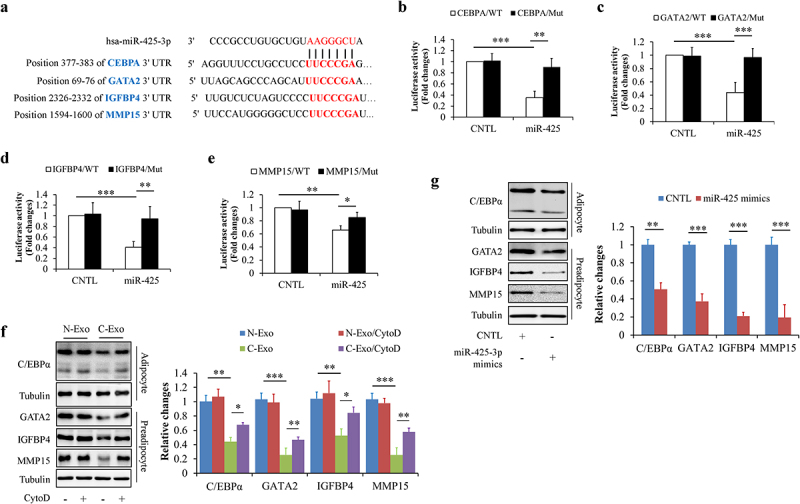
Exosomal miR-425-3p downregulated proliferation- and differentiation-related regulating genes. (a) The predicted miR-425-3p binding site in the 3ʹUTR of the related genes. (b-e) Luciferase reporter tests of miR-425-5p and the related genes. In order to observe the effect of exosomal miR-425-3p on protein levels, HPA-v cells were incubated with 50 μg/L of exosomes or 50 nM of miR-425-3p mimics for 24 h. CytoD (2 μg/mL) was administrated to inhibit endocytosis. (f) Effect of exosomes on the protein levels of C/EBPα, GATA2, IGFBP4, and MMP15. (g) Effect of miR-425-3p mimics on the protein levels of C/EBPα, GATA2, IGFBP4, and MMP15. Data are presented as mean ± SD, n = 4; *p < 0.05, **p < 0.01, ***p < 0.001 *vs* indicated group. N-Exo: normal (NL20) cell-derived exosomes; C-Exo: cancer (A549) cell-derived exosomes; CytoD: cytochalasin D; CNTL: control; WT: wild type; Mut: mutant.

TargetScan online software predicts that some proliferation- or differentiation-related genes may be the potential targets of miR-425-3p, including cell-proliferation regulating genes *GATA2, IGFBP4*, and *MMP15* [16–18], and adipocyte differentiation regulating gene *CEBPA* [19] (Figure 3(a)). Using luciferase reporter assay, we confirmed the association between miR-425-3p and these genes (Figure 3(b-e)). Meanwhile, we found that C-Exo significantly down-regulated the protein levels of GATA2, IGFBP4, MMP15, and C/EBPα in HPA-v cells, which were mitigated by CytoD supplementation (Figure 3(f)). Since exogenous miR-425-3p mimics displayed the same impacts as C-Exo (Figure 3(g)), we thought that exosomal miR-425-3p contributes to C-Exo-reduced protein expressions of GATA2, IGFBP4, MMP15, and C/EBPα in HPA-v cells.

Taken together, these results indicate that cancer cell-derived exosomal miR-425-3p inhibits preadipocyte proliferation and differentiation through targeting related regulating genes such as *GATA2, IGFBP4, MMP15*, and *CEBPA*.

#### Exosomal miR-425-3p promoted adipocyte lipolysis and browning of white adipocyte

**Figure 4.**
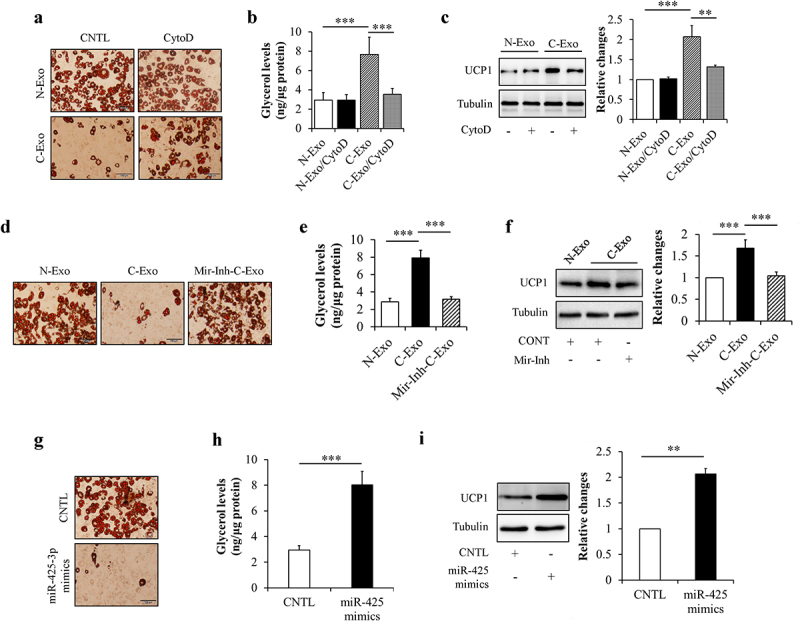
Exosomal miR-425-3p promoted adipocyte lipolysis and white adipocyte browning. Mature adipocytes were incubated with 50 μg/L of exosomes or 50 nM of miR-425-3p mimics for 24 h. CytoD (2 μg/mL) was administrated to inhibit endocytosis. (a) Effect of exosomes on oil red O staining. (b) Effect of exosomes on glycerol concentration in culture medium. (c) Effect of exosomes on UCP1 protein levels. (d) Effect of exosomes with miR-425-3p inhibition on oil red O staining. (e) Effect of exosomes with miR-425-3p inhibition on glycerol concentration in culture medium. (f) Effect of exosomes with miR-425-3p inhibition on UCP1 protein levels. (g) Effect of miR-425-3p mimics on oil red O staining. (h) Effect of miR-425-3p mimics on glycerol concentration in culture medium. (i) Effect of miR-425-3p mimics on UCP1 protein levels. Data are presented as mean ± SD, n = 4; **p < 0.01, ***p < 0.001 *vs* indicated group. N-Exo: normal (NL20) cell-derived exosomes; C-Exo: cancer (A549) cell-derived exosomes; Mir-Inh-C-Exo: cancer (A549) cell-derived exosomes with miR-425-3p inhibition; CytoD: cytochalasin D; CNTL: control.

Lipolysis enhancement and white adipocyte browning are hallmarks of cancer cachexia-induced adipocyte dysfunction and atrophy [5,9]. Like its effects on preadipocyte proliferation and adipogenesis, adipocyte lipolysis and browning were not affected by the administration of N-Exo and CytoD (Suppl. Figure S1c,d). Therefore, we next observed the effects of exosomal miR-425-3p on these pathophysiological processes, through comparing C-Exo with N-Exo. Mature adipocytes were incubated with 50 μg/L of C-Exo, Mir-Inh-C-Exo, or N-Exo in the presence or absence of 2 μg/mL of CytoD for 24 h. Glycerol concentration in culture medium and browning-related protein expressions were measured respectively. We found that C-Exo treatment markedly enhanced glycerol release from adipocytes (Figure 4(a,b)) and increased UCP1 protein levels in adipocytes (Figure 4(c)). Administration of exogenous miR-425-3p mimics generated the similar impacts as C-Exo did (Figure 4(g-i)). In addition, C-Exo-induced alterations were significantly reversed by CytoD supplementation (Figure 4(a-c)) and the inhibition of miR-425-3p (Figure 4(d-f)). These results suggest that exosomal miR-425-3p augments adipocyte lipolysis and white adipocyte browning.

**Figure 5.**
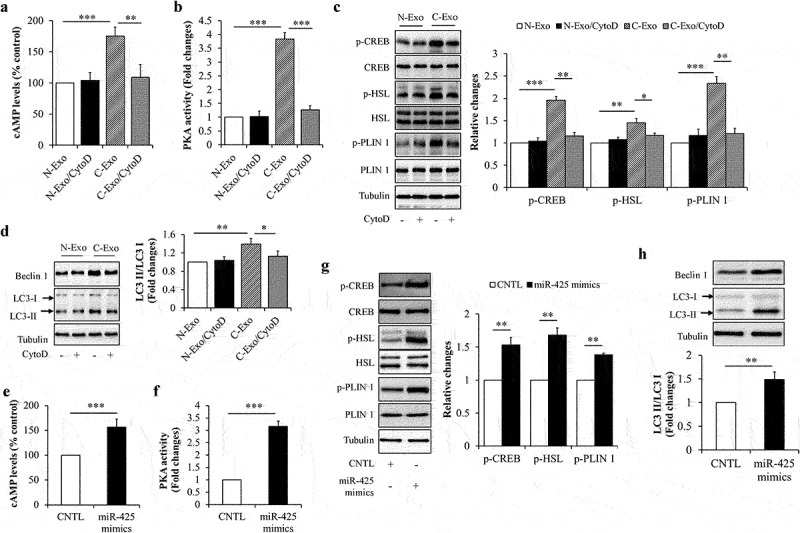
Exosomal miR-425-3p activated cAMP/PKA signalling and lipophagy. Mature adipocytes were incubated with 50 μg/L of exosomes or 50 nM of miR-425-3p mimics for 24 h. CytoD (2 μg/mL) was administrated to inhibit endocytosis. (a) Effect of exosomes on the intracellular cAMP concentration. (b) Effect of exosomes on the intracellular PKA activity. (c) Effect of exosomes on the protein or phosphorylated protein levels of CREB, HSL, and PLIN 1. (d) Effect of exosomes on the protein levels of beclin 1 and LC3. (e) Effect of miR-425-3p mimics on the intracellular cAMP concentration. (f) Effect of miR-425-3p mimics on the intracellular PKA activity. (g) Effect of miR-425-3p mimics on the protein or phosphorylated protein levels of CREB, HSL, and PLIN 1. (h) Effect of miR-425-3p mimics on the protein levels of beclin 1 and LC3. Data are presented as mean ± SD, n = 3; *p < 0.05, **p < 0.01, ***p < 0.001 *vs* indicated group. N-Exo: normal (NL20) cell-derived exosomes; C-Exo: cancer (A549) cell-derived exosomes; CytoD: cytochalasin D; CNTL: control.

To clarify the underlying mechanisms, we observed the effects of exosomal miR-425-3p on cAMP/PKA signalling and lipophagy, both of them have been suggested to play critical roles in the regulation of lipolysis and/or white adipocyte browning [20–22]. We found that both of C-Exo and miR-425-3p mimics could significantly elevate intracellular cAMP concentration (Figure 5(a,e)), enhance PKA activity (Figure 5(b,f)), increase phosphorylation levels of PKA downstream proteins (Figure 5(c,g)), and raise LC3-II/LC3-I ratio (Figure 5(d,h)). Furthermore, C-Exo-induced alterations were markedly mitigated by CytoD treatment (Figure 5(a-d)) and by miR-425-3p inhibition (Suppl. Figure S2). These results indicate that the impacts of exosomal miR-425-3p on lipolysis and white adipocyte browning may be mediated by cAMP/PKA signalling and lipophagy.

### Exosomal miR-425-3p downregulated PDE4B

**Figure 6.**
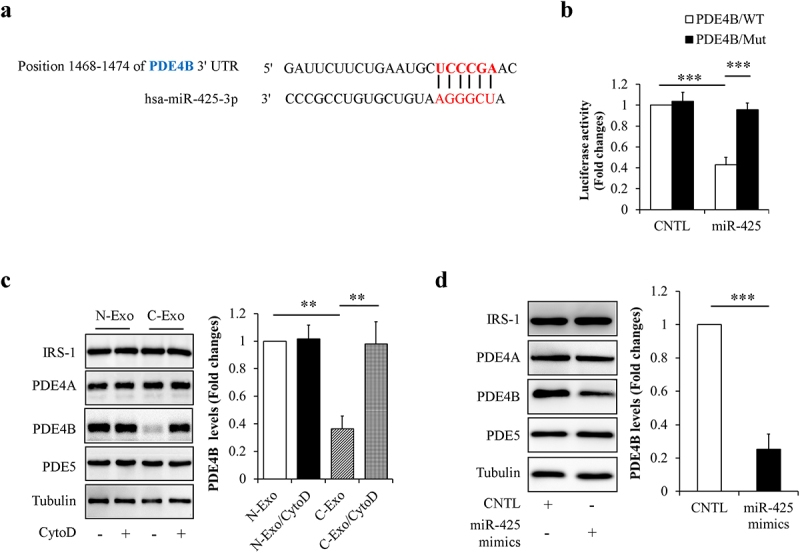
MiR-425-3p targeted phosphodiesterase 4B (PDE4B). (a) The predicted miR-425-3p binding site in the 3ʹUTR of the *PDE4B* gene. (b) Luciferase reporter test of miR-425-3p and *PDE4B*. To observe the potential effect of exosomal miR-425-3p on PDE4B protein levels, mature adipocytes were incubated with 50 μg/L of exosomes or 50 nM of miR-425-3p mimics for 24 h. CytoD (2 μg/mL) was administrated to inhibit endocytosis. (c) Effect of exosomes on the PDE4B protein levels. (d) Effect of miR-425-3p mimics on PDE4B protein levels. Data are presented as mean ± SD, n = 3; **p < 0.01, ***p < 0.001 *vs* indicated group. N-Exo: normal (NL20) cell-derived exosomes; C-Exo: cancer (A549) cell-derived exosomes; CytoD: cytochalasin D; CNTL: control; WT: wild type; Mut: mutant.

PDE4B has been found to highly express in mature adipocytes and to be involved in the regulation of adipocyte function [23,24]. Interestingly, a bioinformatics analysis using TargetScan databases predicts that PDE4B may be a biological target of miR-425-3p (Figure 6(a)). Therefore, PDE4B was chosen for further study. Firstly, we confirmed an association between miR-425-3p and PDE4B by using a luciferase reporter gene assay (Figure 6(b)). Secondly, the effects of exosomal miR-425-3p on PDE4B protein expressions were observed in mature adipocytes treated with C-Exo or miR-425-3p mimics respectively. We found that both of C-Exo or miR-425-3p mimics treatment significantly decreased PDE4B protein levels (Figure 6(c,d)). In addition, inhibition of endocytosis by CytoD abolished the impacts of C-Exo on PDE4B protein expressions (Figure 6(c)). These results demonstrate that PDE4B is a direct target of miR-425-3p.

### PDE4B depletion promoted adipocyte lipolysis and white adipocyte browning

**Figure 7.**
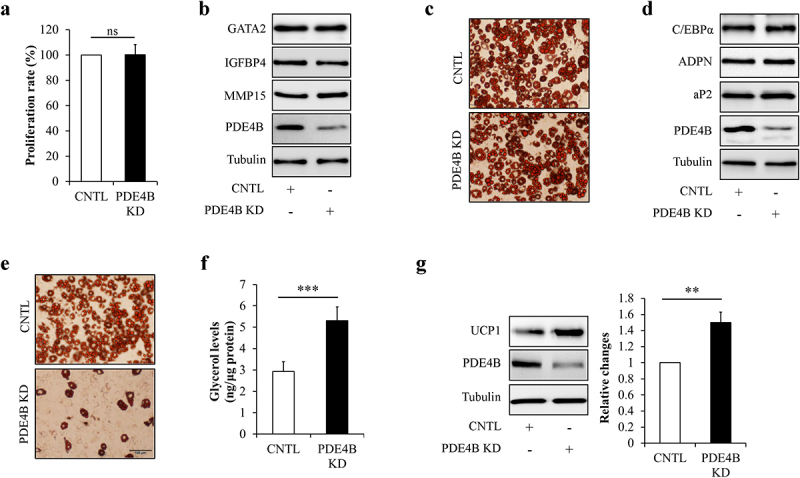
PDE4B depletion did not affect preadipocyte proliferation and differentiation, but promoted adipocyte lipolysis and white adipocyte browning. HPA-v cells were infected with lentiviruses carrying PDE4B shRAN to reduce PDE4B expression. Adipocyte differentiation was induced using a standard protocol. Oil red O staining and western blot were performed at day 7 of adipogenic differentiation. (a) Cell proliferation. (b) The protein levels of GATA2, IGFBP4, and MMP4 in PDE4B KD or control HPA-v cells. (c) Oil red O staining. (d) The protein levels of aP2 and ADPN in differentiated cells. To observe the potential effects of PDE4B on adipocyte lipolysis and white adipocyte browning, lentiviruses carrying PDE4B shRAN were used to knockdown PDE4B in mature adipocyte. (e) Oil red O staining. (f) Glycerol concentration in culture medium. (g) UCP1 protein levels. Data are presented as mean ± SD, n = 4; **p < 0.01, ***p < 0.001 *vs* indicated group. KD: knockdown; aP2: adipocyte protein 2; ADPN: adiponectin; CNTL: control, ns: no significant.

Next, PDE4B was knockdown by infecting HPA-v cells or mature adipocytes with lentivirus/PDE4B shRNA to confirm the potential role of PDE4B in regulating preadipocytes proliferation, adipocyte differentiation, and adipocyte function and so on. We found that cell proliferation (Figure 7(a)), expressions of the proliferation-related regulating genes (Figure 7(b)), oil red O staining (Figure 7(c)), and levels of marker proteins of adipocyte differentiation (Figure 7(d)) remained unchanged in PDE4B-KD cells when compared with control cells, suggesting that PDE4B depletion did not affect preadipocyte proliferation and differentiation. However, knockdown of PDE4B in mature adipocytes significantly decreased lipid contents (Figure 7(e)), increased glycerol release from adipocytes (Figure 7(f)), and raised UCP1 protein levels in adipocytes (Figure 7(g)), suggesting that PDE4B depletion facilitates adipocyte lipolysis and white adipocyte browning.

### PDE4B depletion activated cAMP/PKA signalling and lipophagy

**Figure 8.**
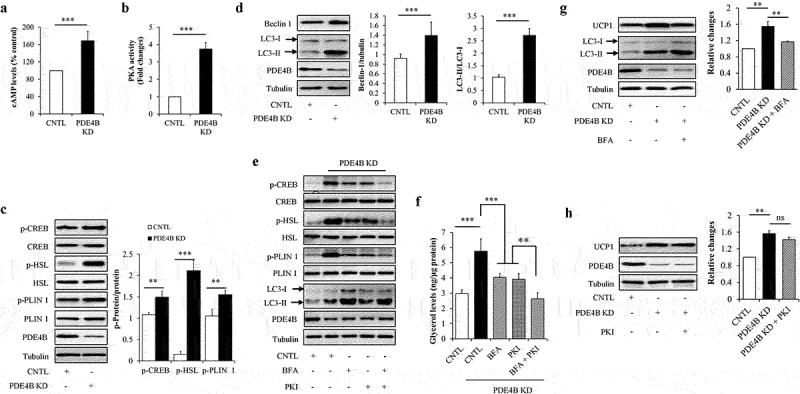
PDE4B depletion enhanced cAMP/PKA signalling and lipophagy. Mature adipocytes infected with lentiviruses carrying PDE4B shRAN or control were treated with or without 100 nM of autophagic inhibitor bafilomycin A1 (BFA) and/or 1 μM of PKA inhibitor PKI for 24 h. (a) Effect of PDE4B depletion on the intracellular cAMP concentration. (b) Effect of PDE4B depletion on the intracellular PKA activity. (c) Effect of PDE4B depletion on the protein or phosphorylated protein levels of CREB, HSL, and PLIN 1. (d) Effect of PDE4B depletion on the protein levels of beclin 1 and LC3. (e) Effects of BFA and PKI on cAMP/PKA signalling and lipophagy in PDE4B KD cells. (f) Effects of BFA and PKI on glycerol concentration in culture medium in PDE4B KD cells. (g) Effect of BFA on lipophagy and UCP1 protein levels in PDE4B KD cells. (h) Effect of PKI on UCP1 protein levels in PDE4B KD cells. Data are presented as mean ± SD, n = 3; *p < 0.05, **p < 0.01, ***p < 0.001 *vs* indicated group. KD: knockdown; CNTL: control; ns: no significant.

Since that PDE4B was a direct target of miR-425-3p and that cAMP/PKA signalling and lipophagy mediated the effects of exosomal miR-425-3p on adipocyte lipolysis and white adipocyte browning, the potential impacts of PDE4B on cAMP/PKA signalling and lipophagy were investigated in mature adipocytes. Intracellular PDE4B was knockdown by infecting mature adipocytes with lentivirus/PDE4B shRNA. We found that PDE4B depletion significantly increased intracellular cAMP concentration (Figure 8(a)), enhanced PKA activity (Figure 8(b)) and phosphorylation levels of its downstream proteins (Figure 8(c)), and raised the LC3-II/LC3-I ratio (Figure 8(d)), accompanied with increased lipolysis (Figure 8(f)) and UCP1 protein levels (Figure 7(g)), suggesting that PDE4B depletion activates cAMP/PKA signalling and lipophagy leading to lipolysis and white adipocyte browning. It was worth to note that combined treatment with autophagic inhibitor BFA and PKA inhibitor PKI markedly reduced glycerol release to normal levels, when compared with BFA or PKI treatment alone (Figure 8(e,f)), suggesting that PDE4B regulates adipocyte lipolysis through both cAMP/PKA signalling and lipophagy. However, PDE4B depletion-enhanced UCP1 protein expressions were only mitigated by BFA treatment (Figure 8(g)) but not by PKI treatment (Figure 8(h)), suggesting that the regulation of PDE4B on white adipocyte browning is dependent on lipophagy, independently on cAMP/PKA signalling.

## Discussion

Although the actual mechanisms remain to be established, growing evidence has suggested that adipose atrophy induced by tumour-derived factors in cancer cachexia might mainly attribute to the alterations of white adipocyte metabolism, including enhancement of lipolysis, inhibition of adipocyte development and lipid deposition (adipogenesis), and browning or beige of white adipocytes, etc [5,25,26]. In the present study, we demonstrated experimentally that cancer cell-derived exosomal miR-425-3p blocked preadipocyte proliferation, inhibited adipogenic differentiation, and promoted adipocyte lipolysis and browning of white adipocytes through targeting proliferation- and differentiation-related genes and *PDE4B*, respectively.

Several studies have revealed the association between miR-425-3p and cancers. In gastric cancer cells, down-regulation of miR-425-3p inhibits immune escape, suppresses migration, and promotes apoptosis [27], suggesting a promotive effect of miR-425-3p on the development and progression of gastric cancer. Most importantly, plasma miR-425-3p and circulating exosomal miR-425-3p levels have been reported to be significantly increased in the patients with lung adenocarcinoma or NSCLC [14,28]. Furthermore, high circulating exosomal miR-425-3p levels are tightly linked to poor progression-free survival in the patients with NSCLC [14]. Therefore, circulating exosomal miR-425-3p is considered as a potent predictive biomarker for low responsiveness to platinum-based chemotherapy and might also represent a promising therapeutic target for therapy-resistant NSCLC [14,29]. In the present study, we found that miR-425-3p highly expressed in both cancer cells and cancer cell-derived exosomes (Figure 1), indicating a possibility of that high circulating exosomal miR-425-3p in the patients with NSCLC may originate from tumour cells *per se*.

Adipose tissue mass is predominantly determined by the formation of new adipocytes from precursor cells (or preadipocytes) and the volume of adipocytes [30,31]. The former is controlled by preadipocyte proliferation and differentiation while the latter usually reflects the balance between lipolysis and lipid storage in adipocytes. It has been documented that preadipocyte proliferation and adipogenic differentiation are complex cellular processes governed by multiple genes such as *IGFBP4, GATA2, MMP15*, and *CEBPA* [16,19,32,33]. Surprisingly enough, these genes were identified as direct downstream targets of miR-425-3p (Figure 3). Silencing these genes by cancer cell-derived exosomal miR-425-3p could undoubtedly inhibit preadpocyte proliferation and differentiation (Figure 2).

It is widely recognized that adipocyte lipolysis is regulated by cAMP/PKA signalling and lipophagy, a selective autophagy that breaks down lipid through a lysosomal lipolytic pathway [20,34]. In this study, both cancer cell-derived exosomal miR-425-3p and PDE4B depletion similarly stimulated cAMP/PKA signalling and lipophagy in mature adipocyte, leading to adipocyte lipolysis (Figure 4, 5, 7). In addition, PDE4B was a direct downstream of miR-425-3p (Figure 6) and PDE4B depletion-stimulated lipolysis could be almost completely suppressed by a combined treatment of autophagic inhibitor BFA and PKA inhibitor PKI (Figure 8(a-f)), suggesting that PDE4B mediated the regulation of exosomal miR-425-3p on adipocyte lipolysis through regulating both cAMP/PKA signalling and lipophagy.

PDE4 is a family of enzymes containing four subtypes (PDE4A, PDE4B, PDE4C, and PDE4D), among them PDE4B is a predominant isoform expressed in adipocytes [23,24,35]. Intracellular PDE4s hydrolyse cAMP and limit stimulation of lipolysis through suppressing PKA activity [35]. Previous study has shown that PDE4B knockout (PDE4B^−/−^) mice exhibits lower body and fat pad weights, smaller adipocytes, decreased serum leptin levels as well as reduction of TNF-α mRNA levels and macrophage infiltration in white adipose tissues on high-fat diets [24], suggesting a strong effect of PDE4B on the function and structure of white adipocytes or adipose tissues. In addition, pharmacological inhibition of PDE4B significantly increases basal lipolysis and reverses in part the antilipolytic effects induced by prostaglandin E2 and phenylisopropyl adenosine in rat adipocytes [35], further confirming the regulation of PDE4B on adipocyte lipolysis. Consistent with these findings, our results evidenced that PDE4B depletion stimulated cAMP/PKA signalling (Figure 8(a-c)) and increased adipocyte lipolysis (Figure 7(e,f)).

In this study, PDE4B depletion has also been found to stimulate autophagy activity (Figure 8(d)). Although mechanism is unknown, the positive relationship between PDE4B knockdown and autophagy activation has been observed in SH-SY5Y neuroblastoma cells [36]. Actually, cAMP can activate autophagy through ERK/cyclin E/Beclin 1 signalling pathway [37]. On the other hand, PKA-dependent lipolysis may directly or indirectly inhibit mTOR, a key negative regulator of autophagy [38,39]. Inhibition of mTOR will result in activation of autophagy [38,39]. Therefore, it seems reasonable to speculate that PDE4B depletion activated lipophagy attributing to its positive regulation on cAMP/PKA signalling or PKA-dependent lipolysis. In addition, lipophagy and lipolysis might create a vicious cycle to facilitate each other. Future *in vitro* and *in vivo* studies are necessary to identify their relationship particularly in the development and progression of cancer cachexia.

It has been well-known that browning of white adipocytes enhances lipid mobilization and the utilization of cellular fuel, contributing at least in part to adipose loss [7,40]. UCP1 is strongly induced during browning processes of white adipocytes and thus recognized as a ‘browning’ marker. In the present study, both cancer cell-derived miR-425-3p (Figure 4(c,f)) and PDE4B depletion (Figure 7(g)) significantly increased UCP1 protein levels, indicating their positive regulations on adipocyte browning. It is noteworthy that the effects of PDE4B depletion on UCP1 could be reversed by autophagic inhibitor BFA (Figure 8(g)) but not by PKA inhibitor PKI (Figure 8(h)), suggesting that lipophagy seems necessary for browning of white adipocytes [22]. In addition, our results found that elevated UCP1 protein levels were accompanied with increased PGC1α expressions in PDE4B-depletion adipocytes (Data not shown). The similar alterations were observed in C-Exo- or miR-425-3p mimics-treated adipocytes (Data not shown). Interestingly, previous study has also confirmed an increased PGC1α levels in the liver of PDE4B-inhibited mice [41]. Since PGC-1α can determine the basal levels of UCP1 in white adipose tissue [42], there is another possibility of that exosomal miR-425-3p regulates browning of white adipocytes through a PDE4B/PGC-1α-dependent mechanism. More studies are required to further confirm this hypothesis.

A previous study has reported that miR-425-3p directly targets Akt1 [14], a key player with high cell-type specificity in controlling cell proliferation and differentiation. In mouse embryo fibroblast cells, adipogenic differentiation is mainly modulated by Akt1 but not Akt2 [43]. On the contrary, the proliferation and differentiation of human preadipocytes is not affected by Akt1 knockdown, whereas it is robustly inhibited with Akt2 deficiency, suggesting that Akt2 but not Akt1 is required for these cell processes [44]. In the present study, we found that both C-Exo and miR-425-3p mimics reduced Akt1 protein levels and inhibited insulin-stimulated Akt phosphorylation (Suppl. Figure S3), suggesting exosomal miR-425-3p also targets Akt1 in human adipocytes. However, overexpression of Akt1 in HPA-v cells at a comparable level was ineffective at restoring C-Exo-induced alterations to normal (Suppl. Figure S4), further confirming that functional regulation of exosomal miR-425-3p on proliferation and differentiation of human preadipocytes is independent on Akt1 [44]. Indeed, previous study has evidenced that lipogenic genes remain unchanged upon Akt1 knockdown [44]. In the present study, we also found that exosomal miR-425-3p inhibited insulin-stimulated phosphorylation of Akt Thr308, an insulin-sensitive residue (Suppl. Figure S3). Of note, the ratio of p-Akt1 T308/total Akt1 remained unchanged, when comparing control group with C-Exo- or mir-425-3p mimics-treated group (Suppl. Figure S3), suggesting that reduction in Akt1 phosphorylation was due to its protein deficiency.

Unlike its effects on adipogenesis, we found that restoration of Akt1 abundance partly prevented against C-Exo-induced alterations in lipolysis and UCP1 expression (Suppl. Figure S5), suggesting that Akt1 is involved in the regulation of these pathophysiologic processes. Indeed, Akt1 has been found to modulate anti-lipolytic effect of insulin [44]. In the present study, we focused on PDE4B-dependent mechanism by which exosomal miR-425-3p controls lipolysis and adipocyte browning. The results do not contradict with the action of miR-425-3p-reduced Akt1 on these cell events. Previous studies have confirmed the interplay between PDE4B and Akt signalling pathways [45,46]. PDE4B inhibition elevates cAMP levels leading to the suppression of Akt activity [45,46]. Considering that ① cAMP/PKA signalling plays a key role in regulating lipolysis [20], ② there is a feedback regulation between autophagy and PKA [47], and ③ Akt is downstream of PDE4B/cAMP signalling [45,46], we thought that the regulation of exosomal miR-425-3p on lipolysis and browning of adipocytes is mainly mediated by PDE4B. However, more studies are required to figure out the crosstalk between PDE4B and Akt signalling pathways, as well as their contribution to lipolysis and adipocyte browning in cancer cachexia.

**Figure 9.**
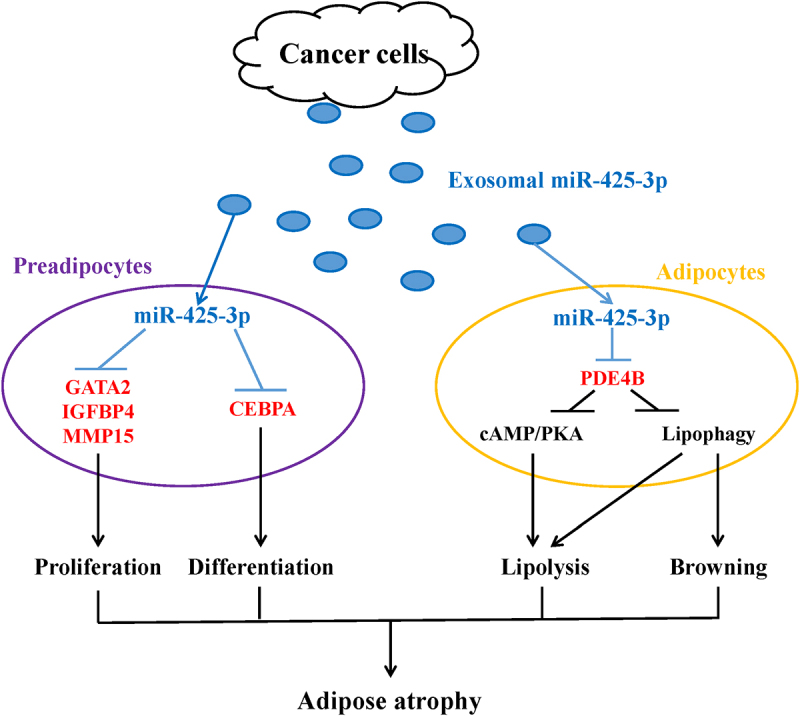
A schematic diagram for the impacts of cancer cell-derived exosomal miR-425-3p on adipocyte atrophy. Cancer cell-derived exosomal miR-425-3p targets proliferation- and differentiation-related regulating genes leading to the inhibition of preadipocyte proliferation and differentiation. In addition, PDE4B downregulation induced by cancer cell-derived exosomal miR-425-3p results in enhancement of adipocyte lipolysis and white adipocyte browning through activating cAMP/PKA signalling and/or lipophagy, respectively. The combined effects of these alterations will bring about adipocyte atrophy, ultimately leading to the loss of adipose tissue in cancer cachexia.

In summary, this study illustrated the effects of cancer cell-derived exosomal miR-425-3p on preadipocyte proliferation and adipogenic differentiation, adipocyte lipolysis, and browning of white adipocytes, and also revealed the potential mechanisms (Figure 9). These findings provide a novel explanation for the development and progression of adipose atrophy in cancer cachexia, although more *in vitro* and *in vivo* studies are acquired to further confirm our findings and to gain a full understanding of its clinical relevance.

## Materials and methods

### Cell culture and adipocyte differentiation

Human preadipocytes-viscereal (HPA-v) cells were obtained from ScienCell Research Laboratories (#7210, Carlsbad, CA, USA). Human non-smallcell lung cancer (NSCLC) A549 cells, human lung carcinoma H1299 cells, human gastric adenocarcinoma AGS cells, human non-tumorigenic bronchial epithelial cell NL20, and human normal gastric epithelial cell GES-1 were all acquired from ATCC (American Type Culture Collection). HPA-v cells were cultured in DMEM containing 5% fetal bovine serum (FBS), 1% preadipocyte growth supplement (#7252, ScienCell Research Laboratories), and 1% penicillin/streptomycin (P/S). Adipocyte differentiation was induced by incubating HPA-v cells with Preadipocyte Differentiation Medium (#7221, ScienCell Research Laboratories) according to the manufacturer’s protocol. Mature adipocytes and other cells were grown in DMEM supplemented with 10% FBS and 1% P/S. All cells were kept in an incubator with a humidified atmosphere containing 5% CO_2_ at 37°C.

### Oil red O staining

Oil red O staining was performed to detect intracellular neutral triglycerides and lipids. Differentiated adipocytes (namely mature adipocytes) were washed with phosphate-buffered saline (PBS), fixed with 10% formaldehyde at room temperature for 20 min, incubated with 60% filtered oil red O stock solution (0.3 g/100 mL in isopropanol) at room temperature for 2 h, and then rinsed with tap water until the water run clear. The images were acquired by a microscope (Olympus, Tokyo, Japan).

### Exosome isolation and treatment

Differential ultracentrifugation was used to isolate and purify exosomes obtained from the culture medium [48,49]. Exosomes were re-suspended in PBS and further confirmed by western blot to detect their marker proteins such as HSP70, TSG101, CD63, and CD81 [49]. Exosome yield was determined by comparing with protein concentration.

To observe the impacts of exosomes on cell function, the cells were cultured in DMEM containing 10% exosome-free FBS, starved of serum for 6 h, and then stimulated with 50 μg/L of exosomes for the desired times [50].

### Determination of cell proliferation, glycerol and cAMP concentrations, and PKA activity

The Cell Proliferation Kit I (#11465007001, Sigma-Aldrich, St. Louis, MO, USA), Free Glycerol Assay Kit (ab65337, Abcam, Cambridge, MA, USA), cAMP Enzyme Immunoassay Kit (CA200, Sigma-Aldrich), and PKA Kinase Activity Assay Kit (ab139435, Abcam) were used to measure cell proliferation, glycerol content in culture medium, intracellular cAMP concentration, and intracellular PKA activity, respectively, according to the instructional guides provided by the manufacturers.

### Virus infection

Lentivirus carrying human PDE4B shRNA (Lentivirus/PDE4B shRNA, sc-44003-V) and Control shRNA Lentiviral Particles-A (Lentivirus/CNTL, sc-108080) were obtained from Santa Cruz Biotechnology (Santa Cruz, CA, USA). Adenoviruses encoding human Akt1 and β-galactosidase (β-gal) were gifts from Dr. Changhua Wang (Wuhan University School of Basic Medical Sciences). β-gal acted as a negative control for Akt1 overexpression. To silence PDE4B or overexpress Akt1, HPA-v preadipocytes or mature adipocytes were starved serum for 4 h, incubated with lentiviruses or adenovirus for another 6 h, and then grown in growth medium for 72 h. The virus-infected cells were selected by 5 μg/mL of puromycin.

### Luciferase reporter assay

Luciferase reporter assay was performed in HEK293 cells as described previously [49]. The results are expressed as the ratio of firefly luciferase activity to Renilla luciferase activity. The sequences of luciferase constructs used to validate the target genes of miR-425-3p were shown in Suppl. Table S1.

### MiR-425-3p quantification and transfection

Total Exosome RNA and Protein Isolation Kit (4478545, Thermo Fisher Scientific, Vilnius, Lithuania) was used to extract and purify total RNA from exosomes. Quantitative real-time reverse transcription-polymerase chain reaction (qRT-PCR) was carried out as described previously [14]. The specific hsa-miR-425-3p primer was 5’-GTACTTCCTGGGATCGGGAATGTCGTGT-3’ [14]. Exogenous ath-miR-156a was considered an external reference. The 2-ΔΔCt method was used for relative quantification of gene expression.

Has-miR-425-3p mimics, inhibitor, and their paired controls were obtained from RIBOBIO (Guangzhou, China). Pre-miR™ miRNA Starter Kit (AM1540, Thermo Fisher Scientific) was used to optimize miRNA mimics or inhibitors transfection into the cultured cells according to the manufacturer’s instructions. Western blot was performed to detect the downstream targets of miRNAs for assessing transfection efficiency at a protein level.

### Western blot

The cells were lysed with a commercial Cell Lysis Buffer (#9803, Cell Signalling Technology, Beverly, MA, USA). The protein concentration was determined using a bicinchoninic acid (BCA) assay. The proteins were separated by a SDS-PAGE gel and transferred onto a nitrocellulose (NC) membrane. Blocking was performed with 5% dry milk at room temperature for 2 h. For antibody staining, the membrane was incubated with primary antibody at 4°C overnight, followed by incubation with secondary antibodies at room temperature for 1 h. Protein bands were detected with an enhanced ECL kit (#32016, ThermoFisher Scientific).

### Statistical analysis

All data are expressed as the means ± standard deviation (SD). Differences between the groups were examined for statistical significance using ANOVA followed by a Tukey *post hoc* analysis. The value of *P* < 0.05 was considered as statistically significant.

## Supplementary Material

Supplemental Material

## Data Availability

Raw data available upon request of senior author.

## References

[1] Roeland EJ, Bohlke K, Baracos VE, et al. Management of cancer cachexia: ASCO guideline. J Clin Oncol. 2020;38(21):2438. 32432946 10.1200/JCO.20.00611

[2] Zhou X, Wang JL, Lu J, et al. Reversal of cancer cachexia and muscle wasting by ActRIIB antagonism leads to prolonged survival. Cell. 2010;142(4):531–543. 20723755 10.1016/j.cell.2010.07.011

[3] Argilés JM, López-Soriano FJ, Stemmler B, et al. Novel targeted therapies for cancer cachexia. Biochem J. 2017;474(16):2663–2678. 28751550 10.1042/BCJ20170032

[4] Argilés JM, Busquets S, Stemmler B, et al. Cancer cachexia: understanding the molecular basis. Nat Rev Cancer. 2014;14(11):754–762. 25291291 10.1038/nrc3829

[5] Daas SI, Rizeq BR, Nasrallah GK. Adipose tissue dysfunction in cancer cachexia. J Cell Physiol. 2018;234(1):13–22. 30078199 10.1002/jcp.26811

[6] Kir S, White JP, Kleiner S, et al. Tumour-derived PTH-related protein triggers adipose tissue browning and cancer cachexia. Nature. 2014;513(7516):100–104. 25043053 10.1038/nature13528 PMC4224962

[7] Petruzzelli M, Schweiger M, Schreiber R, et al. A switch from white to brown fat increases energy expenditure in cancer-associated cachexia. Cell Metab. 2014;20(3):433–447. 25043816 10.1016/j.cmet.2014.06.011

[8] Rohm M, Schäfer M, Laurent V, et al. An AMP-activated protein kinase-stabilizing peptide ameliorates adipose tissue wasting in cancer cachexia in mice. Nat Med. 2016;22(10):1120–1130. 27571348 10.1038/nm.4171

[9] Bing C, Russell S, Becket E, et al. Adipose atrophy in cancer cachexia: morphologic and molecular analysis of adipose tissue in tumour-bearing mice. Br J Cancer. 2006;95(8):1028–1037. 17047651 10.1038/sj.bjc.6603360 PMC2360696

[10] Rydén M, Agustsson T, Laurencikiene J, et al. Lipolysis–not inflammation, cell death, or lipogenesis–is involved in adipose tissue loss in cancer cachexia. Cancer. 2008;113(7):1695–1704. 18704987 10.1002/cncr.23802

[11] Sun X, Feng X, Wu X, et al. Fat wasting is damaging: role of adipose tissue in cancer-associated cachexia. Front Cell Dev Biol. 2020;8:33. 32117967 10.3389/fcell.2020.00033 PMC7028686

[12] Nicolini A, Ferrari P, Biava PM. Exosomes and cell communication: from tumour-derived exosomes and their role in tumour progression to the use of exosomal cargo for cancer treatment. Cancers (Basel). 2021;13(4):822. 33669294 10.3390/cancers13040822 PMC7920050

[13] Mohammadi S, Yousefi F, Shabaninejad Z, et al. Exosomes and cancer: from oncogenic roles to therapeutic applications. IUBMB Life. 2020;72(4):724–748. 31618516 10.1002/iub.2182

[14] Yuwen D, Ma Y, Wang D, et al. Prognostic role of circulating exosomal miR-425-3p for the response of NSCLC to platinum-based chemotherapy. Cancer Epidemiol Biomarkers Prev. 2019;28(1):163–173. 30228154 10.1158/1055-9965.EPI-18-0569

[15] Zhang G, Liu Z, Ding H, et al. Tumor induces muscle wasting in mice through releasing extracellular Hsp70 and Hsp90. Nat Commun. 2017;8(1):589. 28928431 10.1038/s41467-017-00726-x PMC5605540

[16] Bou M, Montfort J, Le Cam A, et al. Gene expression profile during proliferation and differentiation of rainbow trout adipocyte precursor cells. BMC Genomics. 2017;18(1):347. 28472935 10.1186/s12864-017-3728-0 PMC5418865

[17] Maridas DE, DeMambro VE, Le PT, et al. IGFBP4 is required for adipogenesis and influences the distribution of adipose depots. Endocrinology. 2017;158(10):3488–3500. 28938423 10.1210/en.2017-00248 PMC5659704

[18] Gómez-Escudero J, Moreno V, Martín-Alonso M, et al. E-cadherin cleavage by MT2-MMP regulates apical junctional signaling and epithelial homeostasis in the intestine. J Cell Sci. 2017;130(23):4013–4027. 29061881 10.1242/jcs.203687 PMC5769589

[19] Darlington GJ, Ross SE, MacDougald OA. The role of C/EBP genes in adipocyte differentiation. J Biol Chem. 1998;273(46):30057–30060. 9804754 10.1074/jbc.273.46.30057

[20] Rogne M, Taskén K. Compartmentalization of cAMP signaling in adipogenesis, lipogenesis, and lipolysis. Horm Metab Res. 2014;46(12):833–840. 25247872 10.1055/s-0034-1389955

[21] Kloska A, Węsierska M, Malinowska M, et al. Lipophagy and lipolysis status in lipid storage and lipid metabolism diseases. Int J Mol Sci. 2020;21(17):6113. 10.3390/ijms21176113 PMC7504288 32854299

[22] Ro SH, Jang Y, Bae J, et al. Autophagy in adipocyte browning: emerging drug target for intervention in obesity. Front Physiol. 2019;10:22. 30745879 10.3389/fphys.2019.00022 PMC6360992

[23] Mitra MS, Chen Z, Ren H, et al. Mice with an adipocyte-specific lipin 1 separation-of-function allele reveal unexpected roles for phosphatidic acid in metabolic regulation. Proc Natl Acad Sci U S A. 2013;110(2):642–647. 23267081 10.1073/pnas.1213493110 PMC3545773

[24] Zhang R, Maratos-Flier E, Flier JS. Reduced adiposity and high-fat diet-induced adipose inflammation in mice deficient for phosphodiesterase 4B. Endocrinology. 2009;150(7):3076–3082. 19359377 10.1210/en.2009-0108 PMC2703511

[25] Bing C, Trayhurn P. New insights into adipose tissue atrophy in cancer cachexia. Proc Nutr Soc. 2009;68(4):385–392. 19719894 10.1017/S0029665109990267

[26] Du G, Zhang Y, Hu S, et al. Non-coding RNAs in exosomes and adipocytes cause fat loss during cancer cachexia. Noncoding RNA Res. 2021;6(2):80–85. 33997537 10.1016/j.ncrna.2021.04.001 PMC8081875

[27] Li H, Zhao C, Zhao H, et al. Elevated linc00936 or silenced microRNA-425-3p inhibits immune escape of gastric cancer cells via elevation of ZC3H12A. Int Immunopharmacol. 2021;95:107559. 33756228 10.1016/j.intimp.2021.107559

[28] Wang Y, Zhao H, Gao X, et al. Identification of a three-miRNA signature as a blood-borne diagnostic marker for early diagnosis of lung adenocarcinoma. Oncotarget. 2016;7(18):26070–26086. 27036025 10.18632/oncotarget.8429 PMC5041965

[29] Ma Y, Yuwen D, Chen J, et al. Exosomal transfer of cisplatin-induced miR-425-3p confers cisplatin resistance in NSCLC through activating autophagy. Int J Nanomedicine. 2019;14:8121–8132. 31632022 10.2147/IJN.S221383 PMC6790351

[30] Kubota N, Terauchi Y, Miki H, et al. PPAR gamma mediates high-fat diet-induced adipocyte hypertrophy and insulin resistance. Mol Cell. 1999;4(4):597–609. 10549291 10.1016/s1097-2765(00)80210-5

[31] Large V, Peroni O, Letexier D, et al. Metabolism of lipids in human white adipocyte. Diabetes Metab. 2004;30(4):294–309. 15525872 10.1016/s1262-3636(07)70121-0

[32] Gealekman O, Gurav K, Chouinard M, et al. Control of adipose tissue expandability in response to high fat diet by the insulin-like growth factor-binding protein-4. J Biol Chem. 2014;289(26):18327–18338. 24778188 10.1074/jbc.M113.545798 PMC4140255

[33] Feinberg TY, Rowe RG, Saunders TL, et al. Functional roles of MMP14 and MMP15 in early postnatal mammary gland development. Development. 2016;143(21):3956–3968. 27633994 10.1242/dev.136259 PMC5117140

[34] Singh R, Xiang Y, Wang Y, et al. Autophagy regulates adipose mass and differentiation in mice. J Clin Invest. 2009;119(11):3329–3339. 19855132 10.1172/JCI39228 PMC2769174

[35] Wang H, Edens NK. mRNA expression and antilipolytic role of phosphodiesterase 4 in rat adipocytes in vitro. J Lipid Res. 2007;48(5):1099–1107. 17267946 10.1194/jlr.M600519-JLR200

[36] Zhong J, Xie J, Xiao J, et al. Inhibition of PDE4 by FCPR16 induces AMPK-dependent autophagy and confers neuroprotection in SH-SY5Y cells and neurons exposed to MPP+-induced oxidative insult. Free Radic Biol Med. 2019;135:87–101. 30818055 10.1016/j.freeradbiomed.2019.02.027

[37] Ugland H, Naderi S, Brech A, et al. cAMP induces autophagy via a novel pathway involving ERK, cyclin E and Beclin 1. Autophagy. 2011;7(10):1199–1211. 21750416 10.4161/auto.7.10.16649

[38] Mullins GR, Wang L, Raje V, et al. Catecholamine-induced lipolysis causes mTOR complex dissociation and inhibits glucose uptake in adipocytes. Proc Natl Acad Sci U S A. 2014;111(49):17450–17455. 25422441 10.1073/pnas.1410530111 PMC4267365

[39] Jung CH, Ro SH, Cao J, et al. mTOR regulation of autophagy. FEBS Lett. 2010;584(7):1287–1295. 20083114 10.1016/j.febslet.2010.01.017 PMC2846630

[40] He Y, Liu RX, Zhu MT, et al. The browning of white adipose tissue and body weight loss in primary hyperparathyroidism. EBioMedicine. 2019;40:56–66. 30528454 10.1016/j.ebiom.2018.11.057 PMC6412009

[41] Avila DV, Barker DF, Zhang J, et al. Dysregulation of hepatic cAMP levels via altered Pde4b expression plays a critical role in alcohol-induced steatosis. J Pathol. 2016;240(1):96–107. 27287961 10.1002/path.4760 PMC4993672

[42] Ringholm S, Grunnet Knudsen J, Leick L, et al. PGC-1α is required for exercise- and exercise training-induced UCP1 up-regulation in mouse white adipose tissue. PLoS One. 2013;8(5):e64123. 23717545 10.1371/journal.pone.0064123 PMC3661446

[43] Yun SJ, Kim EK, Tucker DF, et al. Isoform-specific regulation of adipocyte differentiation by Akt/protein kinase Balpha. Biochem Biophys Res Commun. 2008;371(1):138–143. 18423396 10.1016/j.bbrc.2008.04.029

[44] Fischer-Posovszky P, Tews D, Horenburg S, et al. Differential function of Akt1 and Akt2 in human adipocytes. Mol Cell Endocrinol. 2012;358(1):135–143. 22480544 10.1016/j.mce.2012.03.018

[45] Suhasini AN, Wang L, Holder KN, et al. A phosphodiesterase 4B-dependent interplay between tumor cells and the microenvironment regulates angiogenesis in B-cell lymphoma. Leukemia. 2016;30(3):617–626. 26503641 10.1038/leu.2015.302 PMC4775385

[46] Smith PG, Wang F, Wilkinson KN, et al. The phosphodiesterase PDE4B limits cAMP-associated PI3K/AKT-dependent apoptosis in diffuse large B-cell lymphoma. Blood. 2005;105(1):308–316. 15331441 10.1182/blood-2004-01-0240

[47] Torres-Quiroz F, Filteau M, Landry CR. Feedback regulation between autophagy and PKA. Autophagy. 2015;11(7):1181–1183. 26046386 10.1080/15548627.2015.1055440 PMC4590648

[48] Helwa I, Cai J, Drewry MD, et al. A comparative study of serum exosome isolation using differential ultracentrifugation and three commercial reagents. PLoS One. 2017;12(1):e0170628. 28114422 10.1371/journal.pone.0170628 PMC5256994

[49] Wu Q, Li J, Li Z, et al. Exosomes from the tumour-adipocyte interplay stimulate beige/brown differentiation and reprogram metabolism in stromal adipocytes to promote tumour progression. J Exp Clin Cancer Res. 2019;38(1):223. 31138258 10.1186/s13046-019-1210-3 PMC6537177

[50] Wen Z, Li J, Fu Y, et al. Hypertrophic adipocyte-derived exosomal miR-802-5p contributes to insulin resistance in cardiac myocytes through targeting HSP60. Obesity (Silver Spring). 2020;28(10):1932–1940. 32844579 10.1002/oby.22932

