# Supplementary material for: Cancer cell-derived exosomal miR-425-3p induces white adipocyte atrophy
Source: Adipocyte. 2022 Aug 8;11(1):487–500. doi: 10.1080/21623945.2022.2108558 (PMC9367658; doi:10.1080/21623945.2022.2108558)
Supplement: Supplemental Material [file KADI_A_2108558_SM9003.pdf]

Supplemental Data

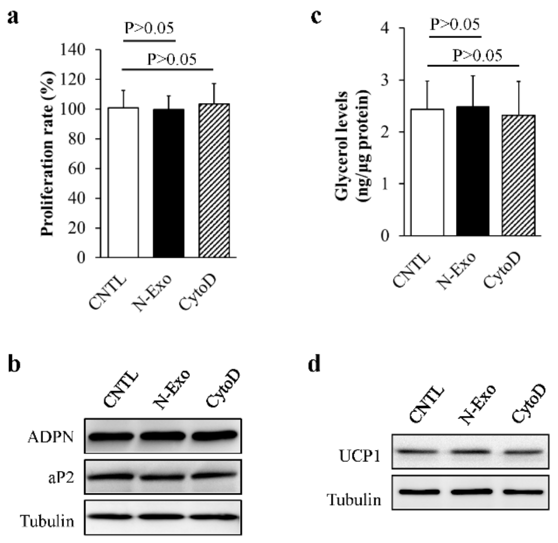

Suppl. Figure 1

Supplemental Figure 1. N-Exo and CytoD did not affect preadipocyte proliferation, adipogenesis, lipolysis, and adipocyte browning. HPA-v cells were incubated with 50 µg/L of exosomes or 2 µg/mL of CytoD for 24 h (for cell proliferation) or 7 days (for adipocyte differentiation), respectively. Adipocyte differentiation was induced using a standard protocol. **(a)** Effects of N-Exo and CytoD on cell proliferation. **(b)** Effects of N-Exo and CytoD on the protein levels of aP2 and ADPN. Mature adipocytes were incubated with 50 µg/L of exosomes or 2 µg/mL of CytoD for 24 h. **(c)** Effects of N-Exo and CytoD on glycerol concentration in culture medium. **(d)** Effects of N-Exo and CytoD on UCP1 protein levels. Data are presented as mean ± SD, n=3. N-Exo: normal (NL20) cell-derived exosomes; aP2: adipocyte protein 2; ADPN: adiponectin; CytoD: cytochalasin D.

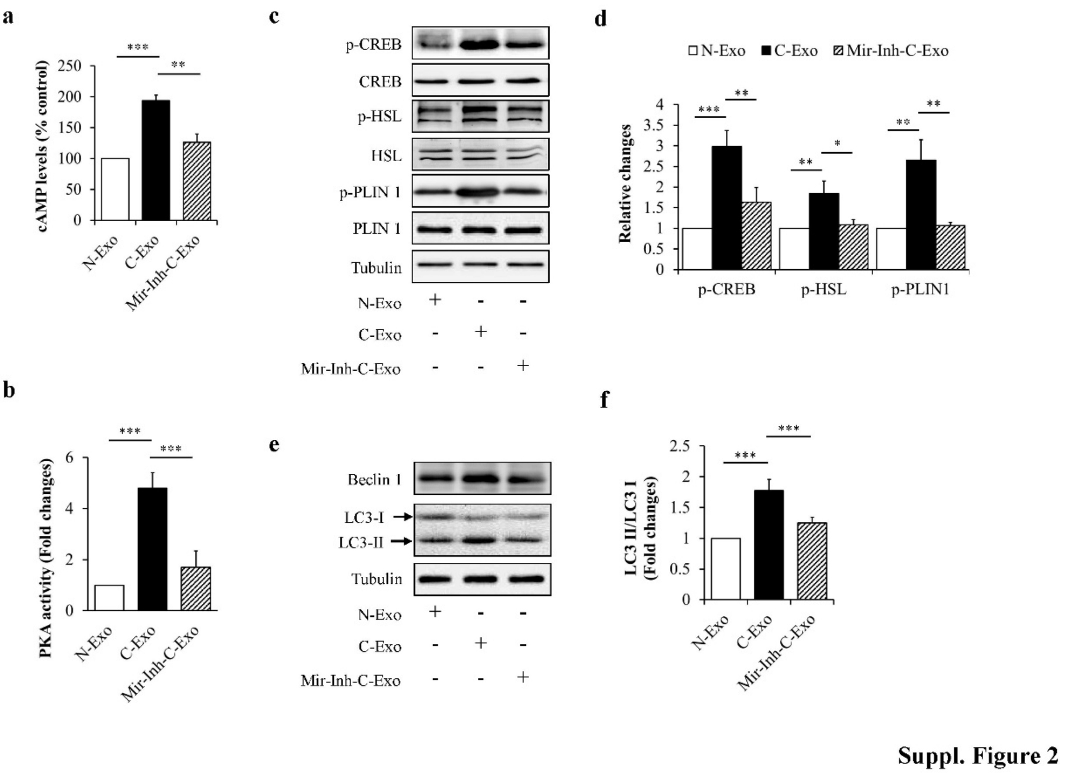

Supplemental Figure 2. Inhibition of miR-425-3p mitigated the effects of C-Exo on cAMP/PKA signaling and lipophagy. Mature adipocytes were incubated with 50 μg/L of exosomes for 24 h. **(a)** Effect of exosomes on the intracellular cAMP concentration. **(b)** Effect of exosomes on the intracellular PKA activity. **(c, d)** Effect of exosomes on the protein or phosphorylated protein levels of CREB, HSL, and PLIN 1. **(e, f)** Effect of exosomes on the protein levels of beclin 1 and LC3. Data are presented as mean ± SD, n=3; **p*< 0.05, ***p*< 0.01, ****p*< 0.001 *vs* indicated group. N-Exo: normal (NL20) cell-derived exosomes; C-Exo: cancer (A549) cell-derived exosomes; Mir-Inh-C-Exo: cancer (A549) cell-derived exosomes with miR-425-3p inhibition.

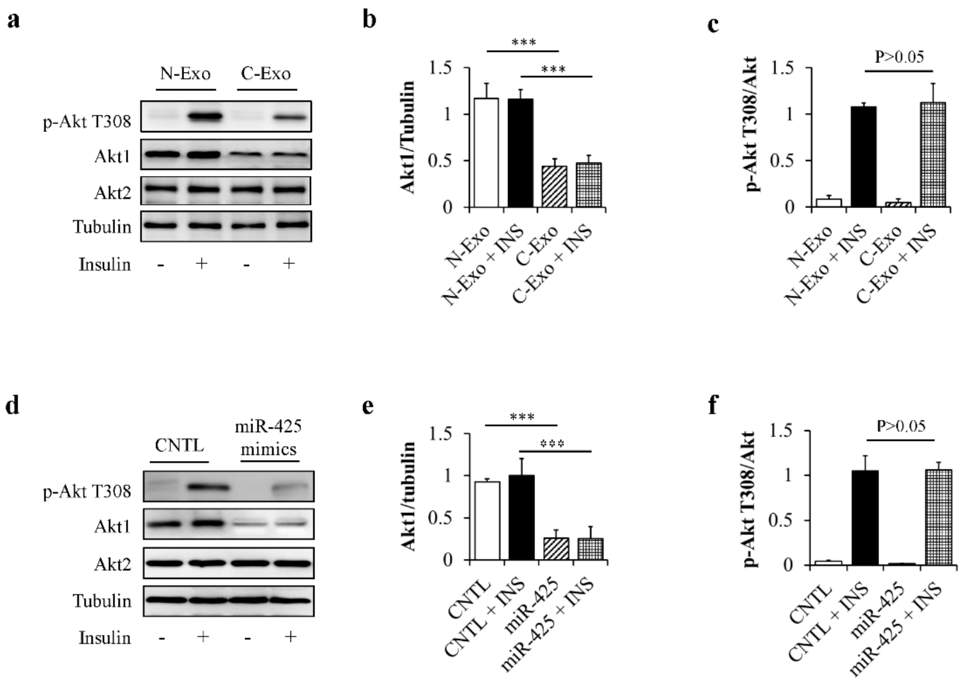

Suppl. Figure 3

Supplemental Figure 3. C-Exo and miR-425-3p mimics suppressed insulin signaling. Mature adipocytes were starved serum for 6 h and then incubated with 50 μg/L of exosomes for 24 h, following by stimulation with 100 nM of insulin for 10 min. **(a)** Effect of exosomes on Akt1 levels and phosphorylation. **(b)** Quantitative analysis of Akt1 protein levels in (a). (**c**) Quantitative analysis of Akt1 phosphorylation in (a). **(d)** Effect of miR-425-3p mimics on Akt1 levels and phosphorylation. **(e)** Quantitative analysis of Akt1 protein levels in (d). **(f)** Quantitative analysis of Akt1 phosphorylation in (d). Data are presented as mean ± SD, n=3; ***$p < 0.001$ *vs* indicated group. N-Exo: normal (NL20) cell-derived exosomes; C-Exo: cancer (A549) cell-derived exosomes; CNTL: control; INS: insulin.

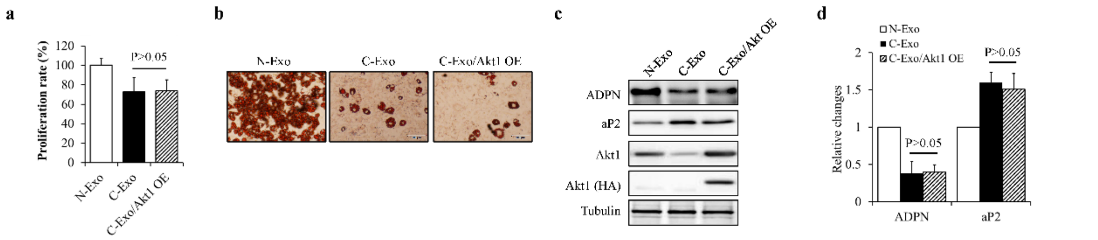

Suppl. Figure 4

Supplemental Figure 4. Akt1 overexpression had no effect on the C-Exo-reduced proliferation and differentiation in human preadipocytes. HPA-v cells were overexpressed with Akt1 and then incubated with 50 µg/L of exosomes for 24 h (for cell proliferation) or 7 days (for adipogenic differentiation), respectively. Adipocyte differentiation was induced using a standard protocol. Oil red O staining and western blot were performed at day 7 of adipogenic differentiation. **(a)** Effect of exosomes on cell proliferation. **(b)** Effect of exosomes on oil red O staining. **(c, d)** Effect of exosomes on the protein levels of aP2 and ADPN. Data are presented as mean ± SD, n=3. N-Exo: normal (NL20) cell-derived exosomes; C-Exo: cancer (A549) cell-derived exosomes; aP2: adipocyte protein 2; ADPN: adiponectin; OE: overexpression.

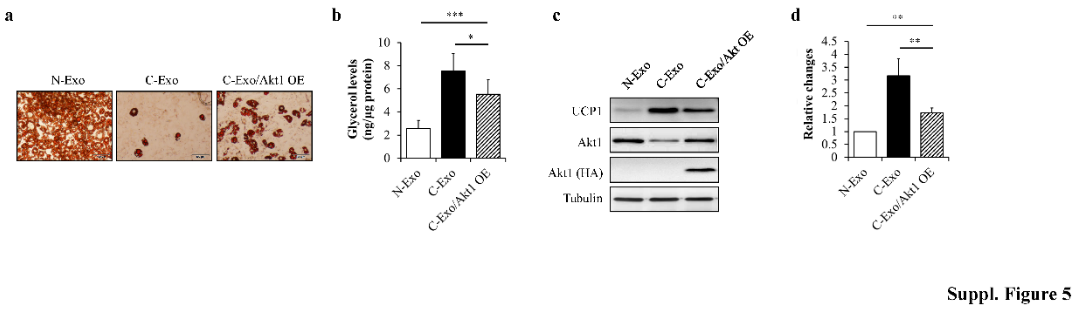

Supplemental Figure 5. Akt1 overexpression partly mitigated the effects of C-Exo on adipocyte lipolysis and white adipocyte browning. Mature adipocytes were overexpressed with Akt1 and then incubated with 50 µg/L of exosomes for 24 h. **(a)** Effect of exosomes on oil red O staining. **(b)** Effect of exosomes on glycerol concentration in culture medium. **(c, d)** Effect of exosomes on UCP1 protein levels. Data are presented as mean ± SD, n=3; *p< 0.05, **p< 0.01, ***p< 0.001 *vs* indicated group. N-Exo: normal (NL20) cell-derived exosomes; C-Exo: cancer (A549) cell-derived exosomes; OE: overexpression.

Supplemental Table 1. Mutations of has-miR-425-3p-binding site

Gene	3' UTR WT	3' UTR Mutant
CEBPA	5'-C**UUCCCGA**G-3'	5'-C**UCAAAGC**G-3'
GATA2	5'-U**UUCCCGA**A-3'	5'-U**UCAAAGC**A-3'
IGFBP4	5'-C**UUCCCGA**U-3'	5'-C**UCAAAGC**U-3'
MMP15	5'-C**UUCCCGA**U-3'	5'-C**UCAAAGC**U-3'
PDE4B	5'-C**UCCCGA**AC-3'	5'-C**UAAAAG**AC-3'
